# Current advances in nanodrug delivery systems for malaria prevention and treatment

**DOI:** 10.1186/s11671-023-03849-x

**Published:** 2023-04-20

**Authors:** Linda N. Kekani, Bwalya A. Witika

**Affiliations:** Department of Pharmaceutical Sciences, School of Pharmacy, Sefako Makgatho Health Sciences University, Pretoria, 0208 South Africa

**Keywords:** Malaria, Nanomaterials, Drug delivery systems, Antimalarial activity, Solubility

## Abstract

Malaria is a life-threatening, blood-borne disease with over two hundred million cases throughout the world and is more prevalent in Sub-Saharan Africa than anywhere else in the world. Over the years, several treatment agents have been developed for malaria; however, most of these active pharmaceutical ingredients exhibit poor aqueous solubility and low bioavailability and may result in drug-resistant parasites, thus increasing malaria cases and eventually, deaths. Factors such as these in therapeutics have led to a better appreciation of nanomaterials. The ability of nanomaterials to function as drug carriers with a high loading capacity and targeted drug delivery, good biocompatibility, and low toxicity renders them an appealing alternative to conventional therapy. Nanomaterials such as dendrimers and liposomes have been demonstrated to be capable of enhancing the efficacy of antimalarial drugs. This review discusses the recent development of nanomaterials and their benefits in drug delivery for the potential treatment of malaria.

## Introduction

Malaria is one of the deadliest diseases in the world and has a high prevalence in subtropical and tropical areas [1]. In 2021, the World Health Organization (WHO) recorded that there were approximately 241 million malaria cases with over 620 000 deaths worldwide in 2020. Africa was reported to be home to 95% of the cases and 96% of malarial deaths, with children under the age of 5 accounting for approximately 80% of the malarial deaths on the continent [2]. This life-threatening, parasitic disease is widely spread by the female Anopheles mosquito and its transmission are high during the hot, wet seasons. These mosquitoes transmit protozoa of the genus *Plasmodium* into the bloodstream of humans. There are over one hundred species of the genus *Plasmodium*, however, only five of these have been shown to infect humans, viz. *P. vivax, P. ovale, P. knowlesi, P. malariae,* and the most prevalent, *P. falciparum*. Although they differ in their drug response, immunology, morphology, and relapse patterns, they can all be prevented, treated, and further managed by the use of artemisinin-based combination therapy (ACT) [2–4].

Two complementary methods are used to prevent malaria: protection against mosquito bites and the use of chemoprophylaxis. Chemoprophylaxis in malaria, also known as chemoprevention, is the use of antimalarial drugs as “protection” from the disease. To protect against mosquito bites, one is advised to use 50% diethyltoluamide (DEET)-based mosquito repellents on the skin, put mosquito nets over their bed, always cover the skin with long pants and sleeves, and use permethrin to treat clothes, tents, and all fabrics [2, 5].

Although malaria is curable and preventable, the disease has not been eradicated due to the parasite becoming resistant to the current artemisinin-based combination therapy drugs. Factors such as the failure to deliver adequate high amounts of drugs to early stages of infected red blood cells, poor patient adherence, the overuse of antimalarial drugs for prophylaxis, and parasite adaptability at genetic and metabolic levels have led to the parasite being resistant to antimalarial therapy [2, 6, 7]. Parasite resistance over the years has forced the scientific community into developing new treatment agents for malaria. Altering the pharmacodynamics and pharmacokinetics of drugs utilizing nanoparticles as an alternative method for drug delivery is getting more attention and is proving to be a viable strategy [8, 9].

Nanoparticles such as liposomes [10, 11], ethosomes [12], hydrogels [13], nanocapsules and nanospheres [14, 15], and nanosponges [16, 17] among others have been functionalized and used in an attempt to circumvent poor patient adherence, parasite resistance, and increase the antimalarial activity of APIs by functioning as API carriers to target drug delivery and enhance API stability. Furthermore, depending on the nature of the nanoparticle, utilizing them as carriers of antimalarial drugs may lead to sustained drug release and high entrapment efficiency. These may result in enhanced parasite clearance and a low rate of recrudescence and a consequent decrease in parasite resistance [18–20].

Herein, we provide a critical review on the current malaria therapy, introduce several types of nanoparticles and provide a critical review of their usage in drug delivery systems, and also investigate their role in the treatment and prevention of malaria.

## Pathogenesis and pathophysiology of malaria

### Pathogenesis

**Fig. 1:**
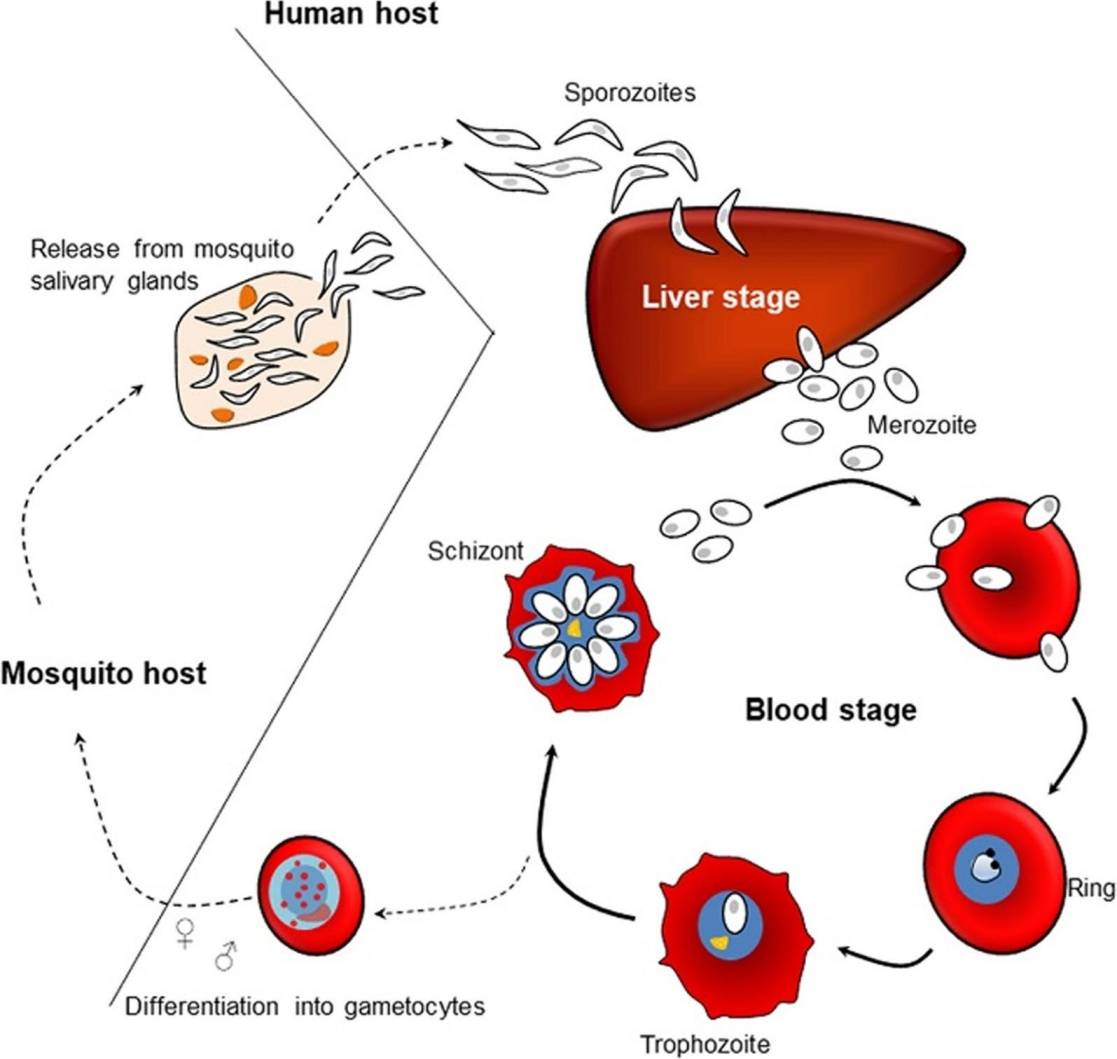
Life cycle of Plasmodium malaria in human vector and mosquito host. Reproduced from [21] in accordance with Creative Commons Attribution License (CC BY 4.0)

Malaria undergoes sexual reproduction in mosquitoes and asexual reproduction in humans. When the infected female Anopheles mosquito bites a human to inoculate tissue parasites, sporozoites are fed into the bloodstream and mark the beginning of the malaria life cycle, illustrated in Fig. 1.

Sporozoites pervade the parenchymal hepatocytes (exoerythrocytic phase) where they multiply asexually and mature into schizonts, which burst to release merozoites into the bloodstream [3]. These invade the erythrocytes, starting the erythrocytic phase. Merozoites have proteins on the surface and apical organelles that protect them from the white blood cells and allow cell recognition and the re-infection of erythrocytes [3, 22].

The erythrocytic phase is a rapid reproducing process powered by glycolysis. In the bloodstream, merozoites synchronously develop into the ring form, trophozoites, schizonts, and merozoites. Merozoites can either re-invade other erythrocytes or further develop into gametocytes (male and female) and undergo sexual reproduction in the vector when the female Anopheles mosquito bites an infected human. This cycle is completed when the mosquito inoculates another human [23, 24].

In the exoerythrocytic phase, sporozoites multiply for 7–28 days. The duration of the erythrocytic phase and the age of infected erythrocyte depend on the *Plasmodium* (*P.*) species and are summarized in Table 1.

**Table 1:** Plasmodium species in different malaria life cycle stages

Period of cycle	*P. falciparum*	*P. vivax*	*P. knowlesi*	*P. malariae*	*P. ovale*
Exoerythrocytic	5.5 days	6–8 days	–	15 days	9 days
Erythrocytic	48 h	48 h	24 h	72 h	48 h
Hypnozoite	No	Yes	No	No	Yes
Ring stage	48 h	48 h	–	72 h	48 h
Invade erythrocytes	All ages	Reticulocytes	Reticulocytes	Old	Reticulocytes
Incubation period	9–12 days	12–17 days or up to 6–12 months	9–12 days	18–40 days	16–18 days
Ability to relapse	No	Yes	Yes	Yes	Yes

Of the *Plasmodium* species known to infect humans and cause disease, *P. malariae* spends the most time, 72 h, while *P. vivax, P. ovale,* and *P. falciparum* all spend 48 h in the erythrocytic stage. Lastly, *P. knowlesi* compared to other species spends the shortest time, approximately 24 h, in the erythrocytic stage [3, 4, 25]. Although *P. malariae* can only invade old erythrocytes, *P. falciparum* can invade erythrocytes of all ages, while *P. vivax*, *P. knowlesi,* and *P. ovale* invade only the young erythrocytes called reticulocytes. Moreover, a certain percentage of *P. ovale* and *P. vivax* species enter a resting stage known as hypnozoite, before undergoing asexual reproduction. The hypnozoite period determines the parasite’s survival and is the cause of multiple relapses [4, 25–28]

### Pathophysiology

The severity and the distribution of malaria are determined by factors such as nutrition, age, immunity, genetics of the host, parasite factors, environmental factors, and the presence of other diseases. Environmental factors such as temperature and humidity affect malaria transmission. According to a study conducted by Mordecai et al. [29], malaria transmission occurs at temperatures between 16 and 34 °C and the optimal temperature of 25 °C. Additionally, it was found that maximum transmission occurs only when humidity increases and the seasonal period of rainfall provides breeding sites for vectors [29–31].

Host factors such as immunity, age, and sex hormones also affect malaria transmission and severity. The sex hormones testosterone and estrogen are said to affect resistance to the *Plasmodium* parasite. However, testosterone weakens the liver’s ability to mount a defense against the parasite and increases the male’s susceptibility to infection, exposing the liver to the parasite and hemozoin for a longer period [32, 33]. In a study by Klein et al., it was discovered that *P. chabaudi* parasites caused higher malaria fatalities in male mice than in female mice. They highlighted that estrogen levels improved immunity and shielded females from disease symptoms when they were infected with malaria [32]. Additionally, in a study by Muehlenbein et al., lower testosterone levels were seen in *P. vivax-*infected men compared to uninfected men of the same age [34]. Although males are more susceptible to malaria than females, testosterone levels decline significantly as parasitaemia increases [35, 36].

Children under the age of five, HIV/AIDS-positive patients, and pregnant women are considered to be at a higher risk. Patients with sickle cell anemia, on the other hand, are said to have some form of conferred immunity from malaria. The sickle-like shaped erythrocytes are permeable and therefore leak nutrients that the parasite needs to live [37, 38].

When the parasites are in the exoerythrocytic phase, there are no clinical symptoms presented. Thus, the pathophysiology of malaria is a result of erythrocyte destruction, as parasitized erythrocytes are responsible for clinical manifestations of malaria. Due to its short erythrocytic phase period, *P. knowlesi* has high parasitaemia. However, *P. falciparum* causes the most severe malaria because of its ability to sequester in microvascular beds throughout the body [4, 39, 40].

### Clinical manifestations

Uncomplicated malaria is defined by the WHO as a “symptomatic malaria parasitaemia with no signs of severity and/or evidence of vital organ dysfunction.” It is determined by variables such as the destruction of parasitized erythrocytes and the intravascular release of host and parasite products at schizogony such as the heme. When this pyretic condition is left untreated, it can progress to severe malaria. Its symptoms are non-specific and mimic those of other diseases [2, 41, 42]. The prodromal symptoms are fever, headaches, muscle aches, malaise, diaphoresis, diarrhea, arthralgia, and abdominal pain. These are usually followed by paroxysmal symptoms caused by the hemolysis of the parasitized erythrocytes, released merozoites, and malaria antigens. Paroxysmal symptoms are grouped into three stages; the cold stage, characterized by rigors and severe pallor, the hot stage characterized by a fever between 40.5 and 41 °C, and thirdly, the sweating stage which lasts for 2–4 h and causes a decline in body temperature. The cold and hot stages last for 15–60 min and 2–6 h, respectively [2, 43, 44].

The WHO defines severe malaria as a “multisyndromic malaria” with clinical and laboratory evidence of vital organ disturbance [2]. This is usually caused by *P. falciparum* and symptoms commonly manifest as respiratory distress, malaria anemia, and cerebral malaria which results in a coma [45]. However, it can also manifest as low blood sugar, significant bleeding, metabolic acidosis, renal failure, hepatic dysfunction, pulmonary edema, hemolysis, focal neurological deficits, multiple convulsions with more than 2 episodes within 24 h, and hypoglycemia [2, 45, 46]. The choice of chemotherapy for malaria is based on the infecting species, patient characteristics such as age, geographic location, and clinical status, and lastly, drug susceptibility of infecting species. Although there is a vaccine available, this determines the type of antimalarial drug that should be administered [2, 47].

### Malaria vaccine

#### Mosquirix (RTS,S/AS01)

In 2021, the WHO recommended utilizing the malaria vaccine for “wide usage” among children in Sub-Saharan Africa and other areas with moderate to high *P. falciparum* transmission [48]. The Mosquirix vaccine, also referred to as RTS,S/AS01 (RTS,S), an intramuscularly delivered lyophilized injection, is a hybrid recombinant pre-erythrocytic stage vaccination intended to inhibit the plasmodium parasite from entering the liver and specifically target the circumsporozoite protein (CSP) by inducing antibodies against it [48–50]. The CSP, a dense protein coat on the surface of sporozoites of the *Plasmodium* species, is necessary for sporozoites to mature [51–53]. Structurally, the CSP has three regions, the N-terminal region, which binds heparin sulfate proteoglycans, the C-terminal region, containing a thrombospondin, and a central repeat region, consisting of a tandem repeat of NANP and accounts for ~ 30% of the entire sequence [52–55].

The malaria vaccine is made up of two components, the RTS,S recombinant antigen and AS01E adjuvant system. The RTS,S antigen consists of the NANP repeats and the C-terminal region of the CSP fused with the hepatitis B virus surface antigen (HBsAg). Herein, the central repeat region of the NANP repeats, represented by “R”, forms a hybrid polypeptide with the T-lymphocyte epitopes separated by immunodominant CD4+ and CD8+ epitopes, denoted by “T”. This generated RT polypeptide is genetically coupled with HBsAg, denoted by “S”. RTS, the recombinant fusion protein, spontaneously combines with free HBsAg to create virus-like particles that give rise to RTS,S [48, 49, 54]. The AS01E adjuvant system composes of liposomes, immunostimulants monophosphoryl liquid A (MPL), and the immunogenic derivative of *Quillaja Saponaria* (QS21) [48, 53, 56–58].

Since B-cell antigens are often said to be proteins with tandem repeat regions, sporozoites get immobilized when antibodies attach to these regions. The RTS,S antigens target B-cell antigenic determinants in the NANP region, which results in humoral responses against CSP [52, 59]. The antibodies of CS repeats, on the other hand, eliminate sporozoites via different mechanisms such as, firstly, cytotoxicity as a result of complement cascade activation. The cascade can be activated to attract and enhance innate immune cells’ phagocytosis, which leads to the destruction of the target cells and shields the host from infection and inflammation [50, 60–62]. Secondly, direct neutralization of sporozoites to prevent cell penetration and migration [50, 63]. Lastly, Fc-receptor mediated lysis and engulfment. These receptors bind to antibodies on the surface of the pathogen and either activate biological activities or inhibit cellular responses by other receptors in the same cell [50, 64].

The WHO reported that the clinical studies for the vaccine were conducted in Ghana, Kenya, and Malawi with over 900,000 children participating in the study in 11 sites [48]. The participants were divided into 2 categories, those aged 5–17 months and 6–12 weeks when the first vaccination was administered. Both categories were given 4 doses in total, however, they were given at different intervals. By the time the participant was 9 months old, they should have received the third dose of the initial 3 doses, which were given monthly. At 15–18 months, the 4th dose had to be administered [48, 49].

White et al. conducted a study to analyze the determinants of immunogenicity after vaccination with or without the booster [65]. They found that participants aged 5–17 months presented with greater RTS,S/AS01- induced anti-circumsporozoite antibody titer after vaccination compared to the other group. However, in the same 5–17-month category, younger participants had a higher titer. Moreover, in participants aged 5–17 months, the RTS,S/AS01 was more immunogenic. Before vaccination, participants aged 6–12 weeks had high baseline anti-circumsporozoite antibody titer and this was associated with low anti-circumsporozoite antibody titer after vaccination. From this, they deduced that immunogenicity is inhibited by either fetal exposure to the malaria parasite or maternal antibodies. Additionally, the efficacy profile of the vaccine for the various categories was obtained at the beginning, at 12 months, and at 5 years. In participants aged 6–12 weeks, the vaccine efficacy was initially 63%, then at 12 months decreased to 11%, and 3% at 5 years.

The booster dose was administered 18 months after the first dose and efficacy increased to 58%, resulting in 8% at 5 years. Vaccine efficacy in participants aged between 5 and 17 months was 74% initially, decreasing to 28% at 12 months, and 9% at 5 years. When the booster was administered 18 months after the first dose, efficacy increased to 59%, then decreased to 17% at 5 years, showing that the typical immunological image of vaccine-induced responses being enhanced to greater levels after primary vaccination does not apply in this instance, which indicates a shorter than usual half-life of memory B-cells.

However, the booster dose was still crucial because the long-lived component of the anti-circumsporozoite antibody response increased from 12% of the post-primary antibody response to 30% of the post-boost response. They also found that for all participants, the vaccine efficacy profile for clinical malaria depended on the intensity of transmission and seasonality at the different sites. In sites with high transmission, efficacy against infection was found to be substantially higher than efficacy against clinical malaria, while in sites with low transmission, the efficacy against clinical malaria was equal to that against infection [65]. In another study, it was also found that the booster dose enhanced vaccine efficacy in both categories and prevented more malarial cases across sites [66].

The vaccination was found to increase the likelihood of febrile seizures within 7 days following the administration of doses 3 and 4, often the first 3 days. However, in phase 3 of the trial, participants who experienced febrile seizures following vaccination fully recovered, and there were no long-term effects. Swelling and pain at the injection site as well as drowsiness, irritability, and fever that subsides a day after immunization are other unfavorable effects. Cerebral malaria and meningitis are regarded as potential risks since there is a low probability that the vaccination may cause them [48, 67–69].

Although the vaccination was shown to decrease the number of participants who were admitted to hospitals and required blood transfusions due to severe malaria, as seen in those who received the booster doses, it is indicated that the vaccine should be used in conjunction with other control measures [66, 69].

#### Other vaccines

Similar to the RTS,S vaccine, the R21 vaccine is also an intramuscularly administered pre-erythrocytic stage vaccine and can likewise elicit anti-CSP antibody responses, but to a larger extent. This is a result of it having a higher CSP content per HBsAg particle. Enhanced efficacy and/or duration of protection might be the result of the improved immune response to CSP, hence it is considered the “next generation RTS,S” [49, 70]. This vaccine unlike the RTS,S vaccine, R21 uses a Matrix-M adjuvant [49, 71]. Matrix-M is a *Quillaja* saponin formulated into nanoparticles with phospholipids and cholesterol, which can activate the innate immune response without the involvement of an antigen while also inducing antibodies of various subclasses [72, 73].

The R21 was tested in Burkina Faso with participants aged 5–17 months. Prior to the malaria season, the participants received 3 doses of the vaccine at 4-week intervals and the 4th dose was administered 1 year later. 28 days following the third dose, they discovered that these participants exhibited significant titers of NANP antibodies specific to malaria. Thus, it was deduced that R21 shows significant high-level efficacy. Although it is still a clinical study, the most commonly noted side effect is fever [70].

## Current treatment outlook

Malaria bouts, according to the WHO, should be treated with at least two drugs to safeguard existing and future antimalarial medicines. As a result, artemisinin derivatives, which are quick acting and have a short half-life, were combined with other long-lasting drugs to develop artemisinin-based combination therapy (ACT). ACT is recommended by the WHO as a first-line treatment for uncomplicated malaria and the high potency, minimal toxicity, and effectiveness against diverse parasite stages of artemisinin and its derivatives puts them at the center of current treatment [41, 74, 75]. ACTs comprise artemisinin derivatives partnered with aryl amino alcohol compounds. These are longer-acting, slowly eliminated antimalarial drugs given over a period of 3 days, viz. halofantrine, lumefantrine, mefloquine, piperaquine, sulphadoxine-pyrimethamine, and amodiaquine. The five ACT combinations have efficacy exceeding 95% and they are: artesunate-mefloquine, artesunate-amodiaquine, artemether-lumefantrine, artesunate-sulphadoxine-pyrimethamine, dihydroartemisinin-piperaquine, the combination of these drugs minimizes the development of drug-resistant parasites [2, 41, 76, 77].

### Artemisinin

*Plasmodium* parasites in erythrocytes reside within a vacuole and digest 70% of the hemoglobin to release heme as a by-product [78, 79]. Since free heme products are harmful to parasites, they are kept as hemozoin polymers in the digestive vacuole. Artemisinin, a sesquiterpene lactone from the plant *Artemisia annua*, has an endoperoxide bridge that breaks when in contact with heme, producing free radicals that are poisonous to the parasite [78, 80, 81]. Although both artemisinin and chloroquine inhibit the synthesis of hemozoin polymers, artemisinin has a shorter parasite clearance time, a half-life elimination of 2–5 h, and acts quicker than other antimalarials. As a result, it is regarded the ideal therapeutic alternative for malaria parasites [2, 74, 82].

Artemisinin derivatives are artemether, artesunate, arteether, and dihydroartemisinin. After being absorbed, they all metabolize into dihydroartemisinin. Dihydroartemisinin acts by releasing free radicals and other reactive metabolites that are harmful to the parasite and by interfering with the parasite’s mitochondrial functions. These derivatives act on various stages of the parasite life cycle, and their elimination half-life varies as well and is summarized in Table 2.

**Table 2:** Properties of antimalarial drugs recommended by WHO

Compound	Solubility	ROA	Active parasite stage	Plasmodium species	Usage	The elimination half-life (hours)	References
Artemisinin	Lipophilic	Oral, rectal	Ring form gametocytocidal	*Falciparum*	Uncomplicated malaria	2–5	[74, 82]
Artemether	Lipophilic	Oral, intramuscular	Schizontocidal	*Falciparum,* *Ovale, Vivax*	Uncomplicated malaria	2–4	[77, 88, 89]
Arteether	Lipophilic	Intramuscular	Schizontocidal	*Falciparum*	Acute and severe malaria	> 20	[83, 84]
Artesunate	Hydrophilic	Oral, parenteral	Schizontocidal Gametocytocidal	*All species*	Uncomplicated And severe malaria	< 1	[76, 77, 83, 86, 87]
Dihydroartemisinin	Hydrophilic	Oral	Schizontocidal	*Falciparum* *Vivax*	Uncomplicated malaria	~ 1	[75, 87]
Quinine	Hydrophilic	Oral Parenteral	Schizontocidal Gametocytocidal	*Falciparum,* *Vivax, Malariae*	Uncomplicated And severe malaria	8–11	[77, 90, 91]
Chloroquine	Hydrophilic	Oral	Schizontocidal	*Malariae, Ovale* *Vivax*	Uncomplicated And severe malaria	20–60 days	[2, 77, 93]
Curcumin	Lipophilic	Oral	Ring form	*Falciparum, Vivax*	Severe malaria	6–7	[97–99]

Dihydroartemisinin has 100% efficiency against malaria and minimal toxicity to humans with an elimination half-life of approximately 1 h. Dihydroartemisinin is a water-soluble drug that is orally administered and is used to treat uncomplicated malaria [2, 75, 83].

Arteether (ethyl ether) is lipophilic, and it is the most stable artemisinin derivative with the longest elimination half-life, > 20 h, and the ability to accumulate in the brain tissue. This is a rapid schizontocidal used to treat chloroquine-resistant severe and acute *P. falciparum* malaria. It is available in two formulations, β-arteether, also known as artemotil, and α,β-arteether [83].

In a study conducted by Looareesuwan et al., artemether and artemotil optimum dose regimens were identical and it was found that artemotil absorption was slower than that of artemether when given intramuscularly. Additionally, another study showed that quinine and arteether have similar outcomes when the route of administration and dose are the same, however, arteether is only restricted for use by children and adolescents under 16 because of the possible effect artemotil might have on the heart [84, 85].

Artesunate (dihydroartemisinin-12-α-succinate) is administered both orally and parentally. It is used for the treatment of both severe malaria and uncomplicated malaria. It has an elimination half-life that is less than 1 h and is both a blood schizontocidal as well as a gametocytocidal drug and is the most commonly used ACT due to its rapid parasiticidal and clinical response. Its combination with mefloquine is extremely effective, even against multidrug-resistant *P. falciparum,* and must be given for a minimum of 3 days [76, 86, 87]. The combination of artemether-lumefantrine, however, is recommended as the first-line therapy of uncomplicated *P. falciparum* malaria by the WHO. It is a short treatment course and is well tolerated [77]. Artemether gives immediate symptomatic alleviation by lowering the number of parasites before lumefantrine eliminates any remaining parasites. Like most artemisinin derivatives, artemether has blood schizontocidal properties, however, its combination with lumefantrine has gametocidal properties [88, 89].

### Cinchona

The *Cinchona* (quina-quina) tree was used to treat fever in the nineteenth century. Continuous testing of the tree revealed that quinine, the active pure compound derived from the bark, could also be used to treat malaria [90]. Quinine is a *Cinchona* alkaloid that is always given as a salt. It is an aryl amino alcohol, just like mefloquine. Furthermore, like artesunate, it is both a blood schizontocidal and a gametocytocidal. It is, however a gametocytocidal against *P. vivax* and *P. malariae* rather than *P. falciparum* [90, 91]*.*

Quinine is a potent antimalarial drug that inserts itself into DNA, disrupting the parasites’ replication and transcription. Albeit its effectiveness against *Plasmodium* species, it is a long-term treatment (> 3 days) with poor tolerability. Cinchonism, bradycardia, digestive problems, and hearing loss are some of its adverse effects. As a result, patient adherence declines, and parasite resistance develops. Although quinine is used for both severe and uncomplicated malaria, it was discontinued as the first-line treatment for malaria due to its risk potential outweighing the therapeutic potential, short half-life, and bitter taste [47, 76, 77, 91, 92].

A quinine derivative, chloroquine, was one of the first widely used and effective antimalarial drugs throughout the world. Chloroquine accumulates in the parasite’s food vacuole and forms a complex with the heme, preventing crystallization. As a result, this inhibits heme polymerase and the build-up of free heme. Compared to other antimalarial drugs, chloroquine has the longest elimination half-life, ranging from 20 to 60 days [93, 94]. Unfortunately, it was overused for chemoprophylaxis, chloroquine-medicated salt, and as insecticide DDT. This influenced the rate at which *Plasmodium* parasites gained resistance against it and increased the incidence of mortality [90, 92]. In addition, the increase in chloroquine-resistant parasites led to chloroquine no longer being used for *P. falciparum* malaria but is still used for *P. ovale, P. vivax, and P. malariae.* The widespread use of these drugs and the tenacity of *Plasmodium* parasites result in the short-term effectiveness of quinine-based drugs [77, 92, 95].

A study by Dondorp et al. showed artesunate was better tolerated than quinine and that quinine had a 7% higher mortality rate when compared to artesunate. For uncomplicated malaria, the WHO advises using oral quinine together with doxycycline only if artemether-lumefantrine is unavailable or contraindicated. Furthermore, if artesunate is not accessible, it is advised for severe malaria since parenteral artesunate has a greater survival rate than parenteral quinine in both children and adults, and intravenous artesunate clears parasites more rapidly than intravenous quinine. This is referred to as a second-line treatment for chloroquine-resistant *P. falciparum* [2, 47, 96]*.*

A summary of the treatment modalities for malaria is provided in Table 2.

### Other plants

There is evidence that suggests that other plant species have the potential to provide anti-malarial moieties. For instance, Teng et al. found that over 1850 plants were reported to be used as antimalarials. Of these, *Cryptolepis sanguinolenta, Annona muricata, Mangifera indica,* and *Carica papaya* were among the most widely used [100]. African herbalists used *Cryptolepis sanguinolenta* to cure malaria without identifying the compounds with antimalarial activity. In a Ghanaian community, Bugyei et al. evaluated the use of *Cryptolepis sanguinolenta* root powder in a 7-day therapy for acute uncomplicated falciparum malaria. This study found a cure rate of 93.5% and instances of recrudescence [101]. Furthermore, *C. sanguinolenta* was compared to chloroquine, but although *C. sanguinolenta* had greater fever clearance, chloroquine had a shorter parasite and fever clearance time. The intrinsic inhibitory activity of cryptolepine and isocryptolepine, alkaloids isolated from the plant’s roots, against *P. falciparum* were evaluated, and cryptolepine proved to be more efficient against the parasite [100–102].

Melariri et al. investigated the effect of different solution extracts on *Carica papaya* leaves*.* They found that *C. papaya* had the highest antimalarial activity when it was extracted using ethyl acetate than water. This extract had the highest selectivity index for the sensitive and resistant strains of *P. falciparum* and the ability to enhance the activities of other component strains with which it was combined. [103]. A study by Bidla et al. showed that the leaves of the Cameroonian plants, *Annona muricata* and *Mangifera indica* exhibited 67% and 50.4% growth inhibition of *P. falciparum *in vitro*.* It was unknown, however, whether this in vitro impact was attributable to the coordinated activity of their components [103, 104].

From the roots of the *Curcuma longa* (turmeric) plant*,* curcumin was isolated. This derivative has been shown to possess antioxidant, anti-inflammatory, and antimalarial activity. [105–107]. In a study by Reddy et al., orally administered curcumin was demonstrated to reduce blood parasitaemia by 80–90% and was said to act as a prophylactic when combined with piperine [105, 107]. Curcumin damages the parasite’s nuclear and mitochondrial DNA, increases the reactive oxygen species which promote the immune response against *Plasmodium* at the trophozoite stage*,* and inhibits the growth of chloroquine-resistant *P. falciparum* [98, 107, 108]. Due to its lipophilic nature, it has a poor dissolution rate, and low bioavailability and its delivery to non-enteric organs is hindered [97, 109]. Curcumin has been shown to have a synergistic effect when combined with artemisinin derivatives and thus can be used against severe malaria [99]. Although various drugs have been discovered and shown to have antimalarial activity, most of these are poorly water soluble and have poor bioavailability.

## Nanodrug delivery systems

**Fig. 2:**
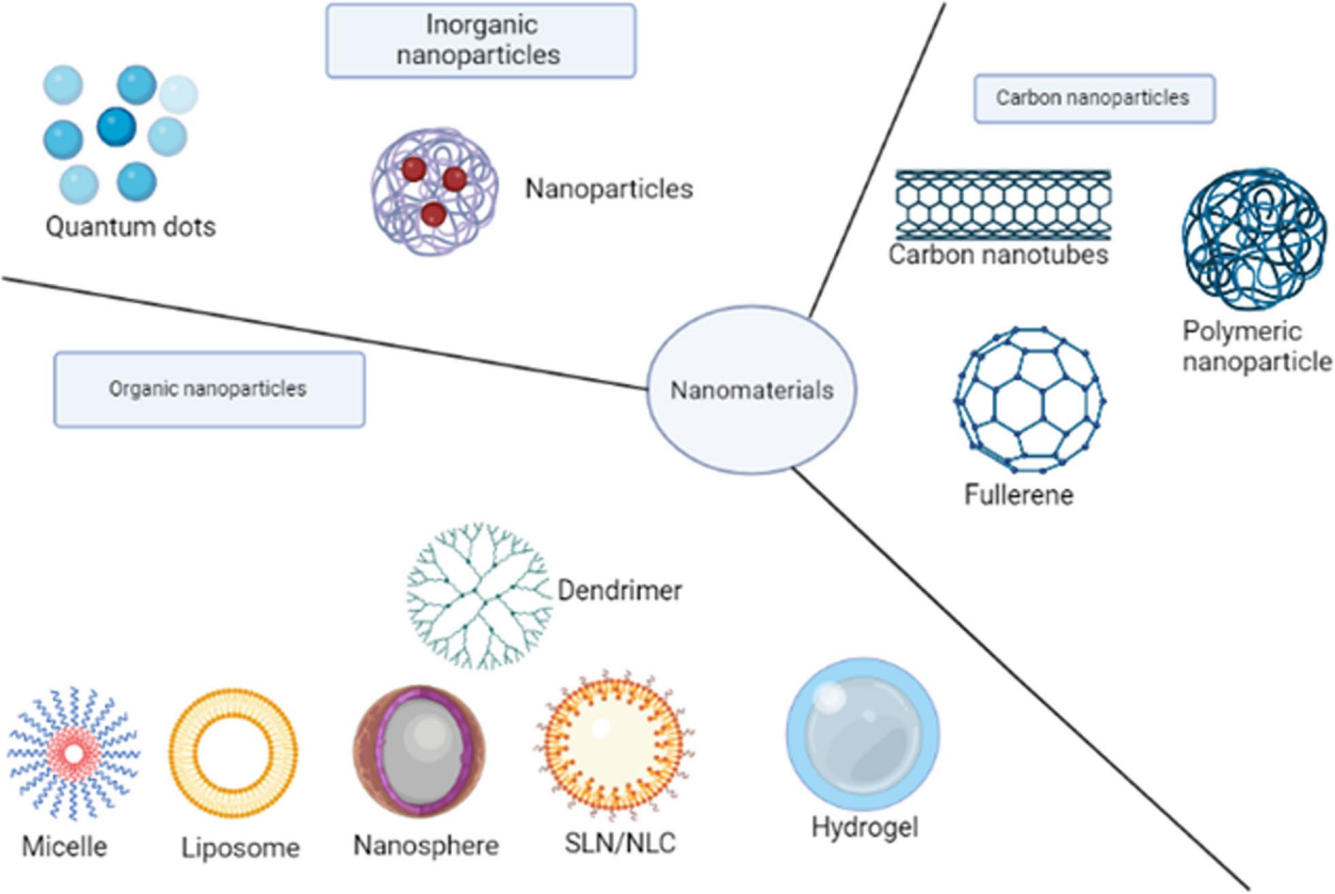
Summary of biocompatible organic nanomaterials used in drug delivery systems for antimalarial drug transport. Reproduced from [119] and Molecular Sciences MDPI in accordance with Creative Commons Attribution License (CC BY 4.0)

Nanoparticles are generally defined as colloidal materials with overall dimensions in the nanoscale and an average size < 1000 nm; however, in nanomedicine the preferred size is < 200 nm [110–113]. Various parameters are used to classify nanomaterials, these include but are not limited to the origin, formation method, and chemical composition. Based on their composition, nanomaterials, shown in Fig. 2, can be categorized into:

- carbon-based nanoparticles, which are entirely composed of carbon atoms such as fullerenes and graphite [114].
- organic-based nanoparticles, which, unlike carbon-based nanoparticles, are created from organic materials other than carbon materials. Examples of these include dendrimers and liposomes [114, 115].
- inorganic-based nanoparticles, such as ceramic metal and semiconductor nanoparticles [116–118].

The colloidal nature of the nanoparticles and their ability to be functionalized allows them to act as drug carriers with target-specific therapeutic properties [120, 121]. Nanoparticles are created using biocompatible biomaterials such as surfactants and/or polymers to construct the encapsulating envelope. Their drug carrier design should address stability in physiological conditions, biocompatibility and biodegradability, minimal toxicity, and high drug loading capacity [122, 123].

Nanoparticles are designed to produce the desired effects in sufficient quantities and yield desired therapeutic responses. Properties such as the surface area to volume ratio, method of preparation, size and shape of the nanoparticles impact the nanoparticle’s behavior, efficacy and mobility via immune system barriers, and must thus be addressed before synthesizing the desired nanoparticles [124, 125]. Physical, chemical, and biological methods can be performed to synthesize nanoparticles in either a top-down or a bottom-up approach. Large, bulk, materials are broken down into nanosized particles using attrition forces in a top-down approach, whereas nanoscale molecules assemble into larger structures [126–128].

Biological synthesis, also called green synthesis, is the synthesis of different metal nanoparticles using bioactive agents such as plants, biowaste, and microorganisms while reducing the consumption of energy and other resources [125]. This process can be scaled up for large-scale synthesis, is cost-effective, simple, produces non-toxic by-products, and yields stable nanoparticles. Here, metallic ions that have undergone reduction or oxidation are used to create nanoparticles in a bottom-up manner [127, 128]. The resulting metallic nanoparticle will have different sizes, contents, shapes and physicochemical properties [129]. These metallic nanoparticles are thought to have the ability to prevent malaria because of their anti-Anopheles larvae properties. Although this method is beneficial, additional research is needed to improve delivery to the target site and preserve particle properties [130–133].

### Lipid-based drug delivery systems

Lipids are defined as esters of fatty acids that are insoluble in water but soluble in organic solvents. These are considered to be one of the most versatile excipients with the potential to control and improve absorption of poorly water-soluble drugs, which are classified by the Biopharmaceutical Classification System (BCS) as class II and IV, in lipid-based drug delivery systems (LBDDS) [134–136].

LBDDS are one of the novel methods used to tackle shortcomings such as the solubility and bioavailability of poorly water-soluble drugs [136, 137]. They are popular because of their ability to potentially protect the drug from the hostile gastrointestinal environment, increase drug payload, reduce toxicity, control delivery to the target site, also and reduce or eliminate the influence of food on the absorption of the drugs [136, 138].

LBDDS can be formulated as lipid solutions, microemulsions, and lipid-drug conjugates (LDC) [135–137]. Formulated LBDDS are characterized by their ability to form oil-in-water (o/w) emulsions due to mild agitation in the gastric fluid. This makes them good candidates for oral delivery of poorly water-soluble drugs. Oral administration of LBDDS is the most popular method as it is cost-efficient, simple, and the most convenient route. However, pulmonary, parenteral, and topical administration routes are also possible 136, 139].

These are usually associated with capsule-filling formulations that are designed to emulsify spontaneously when exposed to aqueous media. These formulations are known as self-emulsifying and self-micro emulsifying drug delivery systems, SEDDS and SMEDDS, respectively 135–137].

### Vesicular drug delivery systems

The self-assembly of amphiphilic building blocks in the presence of water results in the formation of organized assemblies comprised of one or more concentric bilayers which are known as vesicular systems [140]. These are used in drug delivery for the treatment of diseases including malaria, cancer, and pulmonary diseases, among others. They can overcome drug instability, solubility, and rapid degradation due to their properties, which include decreased toxicity, enhanced drug bioavailability and presence in the systemic circulation, and maintained drug release. Vesicular systems enhance patient adherence and efficacy by achieving the long-term therapeutic potential that is highly controlled [10, 140, 141]. There are diverse types of vesicular systems used in the delivery of antimalarial drugs, such as liposomes, ethosomes, and niosomes.

#### Liposomes

Liposomes are spherical, colloidal bilayer vesicles with an enclosed aqueous compartment in the bilayer [11, 142, 143]. Factors such as their hydrophobic (tail) and hydrophilic (head) layers, which allow them to encapsulate and protect materials for delivery to targeted areas, elevated efficacy, and biocompatibility make them popular in drug delivery systems. The lipid bilayer protects the drug from enzymatic degradation while it is in circulation within the extracellular fluid by simultaneously encapsulating hydrophobic materials and attaching the hydrophilic materials in their lumen. Thus, they can be used in targeted oral and topical delivery of ACT drugs [10, 11, 144, [145]. However, small, encapsulated drugs leak in the lumen, and a small fraction of the originally encapsulated drug when membrane fusion occurs delivered. Furthermore, liposomes have a short life, low solubility, are costly and they lack endocytic processes in *Plasmodium-*infected erythrocytes, but they are still able to reduce the risk of toxicity [10, 143, 146].

When lumefantrine was co-loaded with artemether in a liposomal DDS, the technology was found to be stable at 4 °C for 60 days [18]. Additionally, the liposomes were highly absorbed in the spleen and the liver. The in vitro*/*in vivo studies showed no significant evidence of hepatic and renal toxicity. The in vitro drug release study indicated a sustained drug release over a period of 30 h. It was then concluded that liposomes can be used to prolong drug retention in vivo and therefore, the availability of artemether and lumefantrine [18].

In another study, liposomes were used as carriers of artesunate. Although the effect of artesunate liposomes was lower than that of free artesunate after 24 h, the effect of artesunate liposomes stayed the same 72 h after the effect of free artesunate diminished. Suggesting that the liposomes sustained the release of artesunate at the target site [147].

In another study, chloroquine-encapsulating liposomes were injected intramuscularly, intraperitoneally, and subcutaneously into mice. After intraperitoneal injection of liposome-encapsulated chloroquine, the chloroquine concentration in the spleen and liver was found to be higher than that of the free chloroquine after 2–8 h, indicating that the encapsulated chloroquine reached the blood compartments intact and was released gradually. Further comparisons were made between subcutaneously and intramuscularly administered free chloroquine and liposome encapsulated chloroquine. The latter was found to contain a higher chloroquine concentration, indicating a sustained release by the liposome. Although the liposomes acted as “depots” for chloroquine and no toxic effects were found for all the injected liposome encapsulated chloroquine, the intraperitoneal route of administration had the highest blood chloroquine concentration and the muscle depot released faster than the subcutaneous fat layer [148].

#### Niosomes

In the 1970s, Handjanivila and co-workers discovered niosomes. These are non-ionic surfactant vesicles with a bilayer structure and have a diameter size ranging from 10 to 1000 nm. Their bilayer has a hydrophobic head, which is positioned away from the aqueous solvent, and a hydrophilic head, which is positioned toward and is in contact with the aqueous solvent [12, 149].

Niosomes are formed by hydrated self-assembly of non-ionic surfactants and cholesterol. Cholesterol is usually incorporated as an excipient, and this improves membrane rigidity of niosomes making them very stable [149–151]. Factors such as the inclusion of charged molecules, type of encapsulated drug, and type of surfactant not only affect the stability of the niosome but also govern the self-assembly of the surfactant [12, 151]. Although niosomes have properties that render them similar to liposomes, such as their structure, they are both biodegradable, biocompatible, and non-immunogenic, niosomes are cheaper, have a longer shelf-life, more flexible, and more stable 149, 152]. Their stability is attributed to cholesterol and the forces found inside the vesicles. Include van der Waals forces, entropic repulsive forces of the head groups of surfactants, and the repulsive forces from the electrostatic interaction between the charged groups of the surfactants. Moreover, compared to the oily dosage forms, the aqueous vehicle-based suspension formulation of niosomes has superior patient compliance and greater efficacy [149, 153, 154].

Over the years, niosomes have been used to successfully deliver drugs because of their ability to encapsulate hydrophilic, amphiphilic, and lipophilic molecules in their structure [12, 151, 154]. Lipophilic molecules are encapsulated by partitioning into the bilayers’ lipophilic domain, whereas hydrophilic molecules are either entrapped in the bilayer’s inner aqueous core or absorbed on its surface. Niosomes can protect the encapsulated drug from the biological environment, increase drug availability, and so manage the delivery of encapsulated drugs at the target site [12, 151, 154]. Their adaptability in terms of structure and lack of toxicity facilitates easy design for the desired situation. Depending on the type of surfactant used, drug, disease, and anatomical site involved, niosomes can be delivered orally, intraperitoneally, intramuscularly, transdermal, or ocularly [12, 152, 154].

Thakkar and Brijesh developed niosomes co-loaded with Tween 80 and a mixture of primaquine and curcumin then compared it to the individual drugs. Primaquine is encapsulated within the core of the niosomes due to its high solubility in aqueous medium, however, an in vitro release study of the individually loaded niosomes indicated that an initial burst release occurred within an hour and was sustained and was released faster than curcumin. This may be attributed to curcumin’s hydrophobic nature, and the potential restricting role played by the Tween 80 bilayer. The curcumin-loaded niosomes showed a significantly lower suppression than the primaquine-loaded niosomes. Curcumin-primaquine-loaded niosomes showed 100% more antimalarial activity than the other loaded niosomes. In vivo cytotoxicity study found no mortality and the histopathology exam indicated no renal and hepatic toxicity. Overall, the curcumin-primaquine-loaded niosomes had enhanced therapeutic efficacy, increased protection, and survival in rats associated with the prevention of recrudescence. The niosome based curcumin-primaquine therapy was shown to be a promising approach for treating malaria, and it may be due to the greater drug levels at the liver (site of action) [19].

In another study, niosomes were developed as nanocarriers to enhance the antimalarial activity of artemether. The thin-film hydration method containing a mixture of Span, Tween and cholesterol in different molar ratios was used to prepare the niosomes. In vitro drug release study indicated that the formulations of Span 60/Tween 80 released 99% of artemether in 24 h, whereas Span 80/Tween 40 released 87% of artemether in 24 h. The prepared niosomes were relatively smaller in size, measuring ~ 118 nm for Span 60/Tween 80 and ~ 116 nm for Span 80/Tween 40. This was attributable to the surfactant’s hydrophilic-lipophilic balance (HLB), which plays a role in controlling drug entrapment of the vesicle it forms. Moreover, compared to the other niosome, the Span 80/Tween 40 niosome demonstrated a greater entrapment efficacy. This is due to the surfactant’s hydrophobicity, solid nature, and higher-phase transition temperature. The mixture of surfactants entrapped large amounts of small particle artemether molecules. These niosomes had higher artemether activity compared to the free artemether [155].

#### Ethosomes

Ethosomes are described as soft, malleable vesicles containing 20–50% concentrations of ethanol, phospholipids, and water. These non-invasive delivery carriers enable drugs to reach deep skin layers and enhance transdermal absorption of drugs [156, 157]. Ethosomes can be formulated into patches, creams, or gels [158, 159].

Transdermal administration controls drug distribution, bypasses first-pass metabolism and provides enhanced patient compliance. Ethanol in ethosomes is used as a permeation enhancer and allows the vesicle to penetrate the skin. This happens due to the interaction between ethanol and the lipid molecules in the polar head group region, which causes the transition temperature of the lipids in the outermost layer of the skin to drop. This causes an increase in fluidity and a decrease in the density of the lipid multilayer [160, 161].

Artesunate and febrifugine (which has antimalarial activity) were separately loaded into ethosomes. These were incorporated into a matrix of cataplasm to form compound antimalarial ethosomal cataplasm [156]. The artesunate-loaded ethosomes had a particle size of ~ 26.5 nm which was unchanged by the size of artesunate, and encapsulation efficacy of 72%. The febrifugine-loaded ethosomes on the other had a particle size of ~ 28.6 nm and an encapsulation efficacy of 36.3%. These ethosomes were stable at 4 °C for 90 days, and when compared to the conventional cataplasms, they showed enhanced release rates. After 8 h, the accumulative release of artesunate and febrifugine was enhanced by 1.14-fold and 11.86-fold, respectively. The accumulative skin permeation was increased by 1.57 and 1.07-folds for artesunate and febrifugine, respectively. The drug-loaded ethosomal cataplasms had enhanced antimalarial efficiency and recrudescence was fairly insignificant. It was concluded that ethosomal cataplasm could increase the rate at which large amounts of antimalarial drugs rapidly penetrate through the skin [156].

### Solid lipid nanoparticles

Solid lipid nanoparticles (SLN) are submicron-sized (50–1000 nm) colloidal carriers based on melt-emulsified lipids, where the liquid lipids from conventional emulsions are substituted by a solid lipid at room temperature [162, 163]. These emulsions delivery systems consist of physiologically well-tolerated ingredients often generally recognized as safe (GRAS). Their properties include controlled drug release, large surface area, excellent physical stability, and the capacity to avoid untimely drug degradation while immobilizing the drug in the solid matrix. This may ultimately lead to decreased adverse effects and toxicity of drugs [163–165]. These systems have been explored in the delivery of anti-malarial agents.

The use of SLN was investigated for oral administration of arteether. It was found that in arteether-loaded SLN, the payload was protected from degradation by embedding it in the solid lipid matrix, minimizing its exposure to degradation due to the acidic environment in the gastrointestinal tract. This delayed the in vivo metabolism and resulted in arteether having a high entrapment efficacy (~ 69%) [164]. In another study, a combination of artemether and lumefantrine was loaded into the SLN. The in vivo study revealed that the pair has a high parasitaemia clearance after 14 days when administered orally [166].

To evaluate the effect of encapsulation into SLNs on artemether dissolution and permeability in gastric fluids, artemether-loaded SLNs were formulated using high-shear homogenization followed by the ultrasonication method. The formed artemether-loaded SLNs exhibited small particle sizes and high entrapment efficacy [167]. In vitro*,* artemether was released in a biphasic pattern. The first phase was a burst release of artemether during the first 60 min, followed by a gradual release lasting up to 24 h. When compared to free artemether, the encapsulated artemether had enhanced stability and intestinal permeability. This demonstrates that SLNs are a promising approach to overcoming both dissolution and permeability [167].

Chloroquine-loaded SLN and chloroquine-encapsulated heparin functionalized SLN (CH-SLN) were evaluated for activity against *P. falciparum.* The free chloroquine standard had lower antimalarial activity compared to the encapsulated drug. In vitro*,* the CH-SLN and chloroquine-SLN demonstrated 50% and 80% API release after 66 h, respectively. CH-SLN had 50% more efficacy than free chloroquine. From this, it was deduced that SLNs expose the drug to the parasite steadily and this exposure can be maintained over a long period of time [168].

Omwoyo and co-workers developed dihydroartemisinin-loaded SLN to reduce the poor water-solubility of dihydroartemisinin [169]. The formulated SLN was stored at 25 °C for 90 days and there was a significant change in particle size. This increase allowed for the direct absorption of dihydroartemisinin in the gastrointestinal tract and increased its concentration in the liver. Dihydroartemisinin was released in a biphasic pattern, with the initial burst release attributed to an unbound drug on the surface of SLN, which suppresses parasite load. The subsequent gradual release implied that dihydroartemisinin diffused at the release medium. The formulated dihydroartemisinin-loaded SLN released ~ 66% of the payload within the first 5 h, followed by a gradual release lasting up to 20 h, for a total of 96% drug released. Moreover, the efficacy of dihydroartemisinin was enhanced by 25% compared to free dihydroartemisinin, and the encapsulation efficacy was high. The antimalarial activity of dihydroartemisinin was enhanced both in vivo and in vitro*.* From this, it was deduced that dihydroartemisinin-loaded SLNs can be used for drug delivery for clinical application [169].

In another study, a primaquine-loaded SLN was synthesized as a potential delivery system. This was prepared using a solvent emulsification evaporation method based on a water-in-oil-in-water (w/o/w) double emulsification [170]. Chitosan was used as an additive for introducing a positive charge on the surface to increase absorption. Chitosan, a cationic polysaccharide, is a mucoadhesive that aids to enhance circulation time in the intestines, allowing for the absorption of the majority of the nanoparticles. In vitro*,* the payload was gradually released from the SLNs over 72 h with no initial burst release. Chemosuppression of primaquine-loaded SLNs was higher than that of free primaquine at 93.5% and 71.9%, respectively. The data suggest that primaquine encapsulation into an SLN can lead to an increase in antimalarial activity, as evidenced by the nanoformulated technology exhibiting 20% more effectiveness than the standard oral dosage form [170].

Artesunate-loaded SLNs were fabricated using a microemulsion dilution technique to improve the aqueous solubility and oral bioavailability of artesunate. Artesunate release was evaluated at pH 1.2 and 6.8, and it was found that an increase in pH increased the drug release rate. At pH 1.2 and 6.8, the pure artesunate was rapidly released within the first 30 min whereas the artesunate-loaded SLN showed an initial burst release in the first hour and was followed by a sustained release over time. This nanoformulation enhanced the aqueous solubility, bioavailability, and intestinal permeability of artesunate [171].

### Polymeric drug delivery systems

In drug delivery systems, polymers are often utilized because of their ability to control the target release of drugs and adjust the circulatory half-life characteristics of the drug. Drug release from these systems can be controlled by diffusion, the presence of other chemicals, activated solvents, or stimuli [172, 173]. Micelles, polyplexes, hydrogels, and polymer-drug conjugates are examples of responsive polymers for drug delivery and can alter their chemical and/or physical properties in response to external stimuli [172, 174]. Amongst these, micelles are the most commonly used due to their unique hydrophilic shell structure, which may prolong the drug’s presence in the blood circulation and be functionalized with different ligands to alter the micelle permeability and their hydrophobic core which is used to encapsulate poor water-soluble drugs and increase their solubility and be modified to control drug release [172, 173, 175].

In the body, PLA and PLGA are degraded by hydrolysis of their ester-bond backbones. PLGA forms glycolic acid and enters the Krebs cycle to be eliminated as carbon dioxide and water. PLA, on the other hand, degrades into lactic acid or carbon dioxide and water. These products can be further metabolized intramuscularly, excreted in urine, feces, or breathed out [176–179]. Polymer degradation occurs on both the surface and inside the polymeric structure, ideally resulting in the fragmentation of small, non-toxic metabolic compounds. The size and safety of the degraded compounds, however, are uncertain [172, 176].

Musabayane et al. avoided the bitter taste of orally administered chloroquine to increase patient adherence by using a hydrogel chloroquine-pectin patch to be applied on the skin. The chloride-hydrochloric acid was used as a solution to test drug release. The in vitro release study of the patch showed that 30% of chloroquine was released when the solution was at pH 7.4 after 2 h, and 76% was released after 6 h. Additionally, when the solution was at pH 1.0, only 10% was released after 6 h. It was concluded that this patch might have potential applications for the transdermal delivery of antimalarials [180].

Chitosan, a biodegradable polymer, was used to coat magnetic nanoparticles encapsulating artemisinin. Artemisinin loading capacity in chitosan magnetic nanoparticles increased as chitosan concentrations rose. This demonstrated how nanoparticles may be modified to fit the intended application of a formulation. These magnetic chitosan nanoparticles were delivered to the target site, which had greater drug concentrations [181]. Since the chitosan coat has mucoadhesive properties that interact with the mucus layer and increases residence time at the site of absorption, helping to attenuate drug release and obtaining an adequate drug concentration at specific immune system sites, using chitosan-coated nanoparticles may be an alternative to increase the bioavailability of artemisinin [181]. Despite the biocompatibility of the polymeric materials aforementioned, care must be taken when formulating them and they need to be examined further both in vivo and in vitro [122, 172, 182]. Polymeric drug delivery systems such as nanocapsules, nanospheres, and hydrogels have been employed to deliver antimalarial drugs.

#### Nanocapsules and nanospheres

Nanocapsules are defined as vesicular systems, hollow-core structures in which drugs are encapsulated within the central volume surrounded by an embryonic polymeric sheath. Lipids, polymers, and phospholipids can all be used in their preparation [14, 183]. Nanospheres are solid core spherical particulates that contain the drug embedded within the matrix [14, 15]. Preformed polymers can be used to make nanocapsules and nanospheres via interfacial deposition of polymer (containing oil) or nanoprecipitation (without oil in the formulation), respectively. Oil causes a vesicular structure in nanocapsules but a matricial arrangement of the polymeric chains in nanospheres when it is absent [184, 185].

Chloroquine-loaded PLA nanospheres were developed to improve the efficacy of chloroquine. Chloroquine-loaded PLA was prepared using emulsification-solvent evaporation and the nanoprecipitation method. The emulsification-solvent evaporation-prepared loaded PLA showed lower cell viability levels than those observed for free chloroquine at the same concentration range, suggesting that the loaded PLA improved chloroquine activity and its uptake by the cell. Although both preparation methods resulted in increased chloroquine activity, the emulsification method had better encapsulation efficiency and thus, better activity [186].

#### Hydrogels

Hydrogels are described as cross-linked networks of water-soluble polymers. These 3-dimensional networks are produced by the simple reaction of one or more monomers and have a porous structure that permits the encapsulation and release of drugs [13, 187].

Hydrogels can absorb and retain copious amounts of water because of the hydrophilic functional groups found on their backbones. Their degree of porosity is influenced by the composition of the polymer, the nature of the materials used, and the method of preparation. Poly(lactic acid) (PLA) and poly(lactic-co-glycolic acid) (PLGA) are examples of hydrogel-forming polymers. These polyesters offer a secure framework for safely delivering drugs without harming the body since they are biodegradable and bioabsorbable [188–193]. Although PLA and PLGA have similar chemical structures, they release drugs at different rates. PLGA has a faster release rate because it lacks methyl groups found in PLA, resulting in lower water uptake, while still being insoluble in water and being able to absorb water [172, 178].

Aderibigbe and Mhlwatika developed soy protein isolate-carbopol-polyacrylamide-based hydrogels loaded with a combination of chloroquine phosphate and curcumin. The formed hydrogels were pH sensitive. The swelling capacity of the hydrogel can be used to control the rate of release of drugs from the hydrogel and at pH 7.4, the hydrogel exhibited enhanced swelling. This was attributed to the presence of acrylic acid, as hydrogels without acrylic acid exhibited the least swelling. The in vitro single drug release kinetics showed chloroquine diphosphate at pH 1.2 and pH 7.4 was 36.4 and 88.323%, respectively, over 24 h. The release at acidic conditions, pH 1.2, suggested that the hydrogels can be used to deliver drugs to the gastrointestinal tract. In the dual release kinetics, at pH 1.2 and pH 7.4, 33% and 77%, respectively, of chloroquine diphosphate was released over 24 h. The release of curcumin on the other hand was evaluated after 3 days. It was found that 31% and 50% of curcumin was released at pH 1.2 and pH 7.4, respectively. The single release of chloroquine was rapid and lasted a short time, but the dual release of both drugs under the same conditions persisted longer, suggesting that hydrogels are prospective dual drug delivery systems in which both drugs function over different periods to overcome drug resistance [13].

Another study found that single-loaded hydrogels released drugs faster than hydrogels loaded with two drugs [13]. However, because antimalarial drugs are given as combination therapy to prevent drug-resistant parasites, extensive research was conducted. Chloroquine diphosphate and curcumin (water-insoluble antimalarial drug) were both encapsulated onto a soy protein isolate-carbopol-polyacrylamide-based hydrogel. The hydrogels were pH-sensitive, and their swelling capability was enhanced at pH 7.4. The scanning electron microscopy images of the hydrogel showed that the hydrogel lost its morphological properties (swollen and spherical) after drug release in acidic conditions, suggesting that it is biodegradable. The degree of swelling of hydrogels corresponds with the rate of drug diffusion in the hydrogel and is used to control the rate of drug release from the hydrogel. Chloroquine diphosphate had a slower release mechanism at pH 7.4 because it is more water-soluble, while curcumin was released faster. This is due to the ability of hydrophilic functional groups (located at the polymeric backbone of hydrogels) to attract water molecules, which then force them to open. Over 24 h, 77% and 81% of chloroquine diphosphate and curcumin were released at pH 7.4, respectively. The authors concluded that the dual release mechanism of drugs by the hydrogel was influenced by the presence of the other drug in the hydrogel and that hydrogels can be used for the delivery of combination therapeutics [13].

To prolong the gastric residence time of drugs, Liu et al. developed triggerable tough hydrogels. These hydrogels can be ingested, can withstand gastrointestinal motility forces, alter their morphology to ensure prolonged gastric residence, and although they exhibit stability in normal gastric environments, they can be triggered to dissolve rapidly with biocompatible chelators and reducing agents, such as alginate and polyacrylamide, and control the release of drugs. A one-step method, where all the ingredients needed to form the two networks were dissolved in deionized water and heated at 50 °C for 1 h, was used to fabricate the hydrogels. The hydrogels were loaded with lumefantrine. The release profile of lumefantrine from the hydrogen was first order. The in vitro results showed that the release of lumefantrine after a 12-day incubation increased from 8.3% to 61%, and drug loading decreased from 10 to 1%. This suggested that diffusion of the drug decreased when the size of the hydrogel decreased due to the increase in hydrophobicity of the gel associated with lumefantrine. In vivo, the rate constant for lumefantrine release by the hydrogel was 0.68 day^−1^, this was three times higher than the in vitro release rate constant. The hydrogel prolonged the gastric residence and thus delayed its release. This was indicated by the low elimination rate constant of 0.68 day^−1^ for the formulation compared to the 1.17 day^−1^ of the free drug. No significant toxicity was observed and the GITs of the used animals were not affected in anyway [194].

### Nanosponges

Nanosponges, like most drug delivery systems, are biodegradable, have negligible toxicity and are biocompatible. Although they come in a variety of sizes and forms, they are typically between 200 and 300 nm in size [16, 195]. These are cross-linked polymer-based solid colloidal structures. Described as hyper-reticulated nanoporous 3D supramolecules, nanosponges are hydrophilic in nature and feature an inner cavity, that allows the addition of hydrophilic molecules, and an eternal polymeric network that permits them to accommodate fewer lipophilic molecules [16, 17].

Nanosponges can be functionalized for specific site targeting by conjugating different ligands to their surface. Nanospheres can form either inclusion or non-inclusion complexes with molecules and as a result, they may enhance the solubility and bioavailability of drugs. Nanosponges offer a regulated pattern of drug release. Moreover, they can be used as excipients, to mask unpleasant flavors, and to protect the encapsulated molecules from chemical enzyme-induced degradation [16, 17, 196].

There are various types of nanosponges and they are distinguished by the type of method used, the drug they contain, and the type of polymer or cross-linker used [196]. These are: silicon nanosponge particles, carbon-coated metallic, hypercross-linked polystyrene, ethylcellulose based, titanium-based, DNAzyme, and the commonly used β-cyclodextrin based nanosponges [16, 17, 197]. Majority of these nanosponges have been applied for the drug delivery of anticancer agents [16, 17, 198, 199], management and treatment of infectious diseases [200, 201], and other diseases [198, 202]. In the treatment of malaria, cyclodextrin-based nanosponges are the most widely explored and are the focus of this section.

#### Cyclodextrin-based nanosponges

**Fig. 3:**
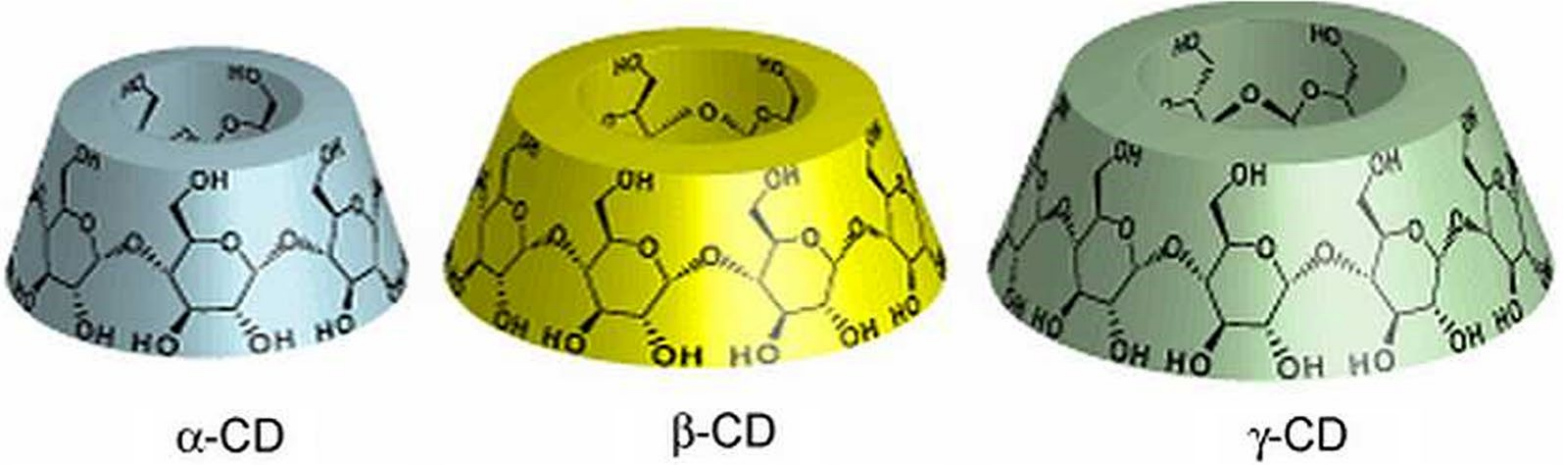
Chemical structure of three types of cyclodextrins: comp Reproduced from [205] in accordance with Creative Commons Attribution License (CC BY 3.0)

Cyclodextrins are naturally occurring cyclic hosts. They have 6–8 d-glucose monomers linked through α-1, 4-glycosidic bonds. The structures are named according to the number of D-glucose monomers available, when the structure has 7 monomers it is called β-cyclodextrin, shown in Fig. 3. When it has 6 and 8 D-glucose monomers it is called α- and γ-cyclodextrin, respectively. Amongst these molecules, β-cyclodextrin is mostly used as a carrier. Cyclodextrins have a hydrophobic interior and a hydrophilic exterior and can encapsulate a hydrophobic drug guest molecule through recognition to form a host–guest complex [203, 204].

Cyclodextrins are cost-efficient and can decrease the clearance of drugs by increasing their stability, solubility, and dissolution rate [206, 207]. Although the cyclodextrin complex of a drug is more hydrophilic than the drug itself, thus increasing the solubility of the drug, it can also affect its chemical characteristics.

For instance, in a study conducted by Yang et al., 2-hydroxypropyl-β-cyclodextrin (HPβCD), a hydroxy alkylated β-cyclodextrin with improved water solubility, was used as a carrier of artemether and this formed a host–guest complex. This host enhanced the water solubility and bioavailability of artemether by 1.81-fold. When taken orally, HPβCD protected the guest molecule by preventing its degradation in the GI tract, releasing it in the intestines where it is absorbed and HPβCD is then excreted in feces [204, [208]. Moreover, it was found that not only did this HPβCD-artemether complex increase the solubility and dissolution of artemether, but it also did not demonstrate any sticking and chipping [209].

Artesunate formed a complex with β-cyclodextrin in the presence of polyethylene glycol (PEG). The β-cyclodextrin complex with artesunate increased the solubility of free artesunate 13.4-fold. Furthermore, when the complex was formed in the presence of PEG, the solubility of the complex increased by 20 times [210].

In another study, primaquine formed a complex with HPβCD. Due to primaquine being a hydrophilic drug, it interacted poorly with HPβCD by binding to the external rim and forming a non-inclusion complex with HPβCD. This complex reduced the rate at which primaquine was released. After complexation, approximately 96% of primaquine was released 21,2 h slower than the free primaquine, and the activity of primaquine was increased [211, 212].

### Pristine nanomaterials

Nanosuspensions also known as nanocrystal suspensions were discovered as a way to overcome poor water solubility of drugs for improved drug delivery. These are nanosized colloidal dispersions of entirely pure drug particles in surfactants. Surfactants act as stabilizers, adjusting the size and shape of nanostructures [163, 213–215]. A liquid media with either a bottom-up method, where basic units assemble into larger structures or a top-down method, where micromolecules are reduced to nanocrystals using attrition forces, are used to form nanosuspensions [126, 216, 217]. The preparation of nanosuspensions is a simple process and can be used for water-insoluble drugs. The advantages of nanosuspensions also include a higher loading capacity, a possible dose reduction, enhanced chemical and physical stability of the drug, and passive drug targeting [215].

Nanosuspensions have enhanced oral and topical formulations of antimalarial drugs. For instance, nanocrystals of artemether were produced using top-down technology (a Dena® reduction machine) to investigate possible changes in the aqueous solubility and antimalarial activity of artemether. A solution of Hypromellose (HPMC), polyvinylpyrrolidone (PVP)-K30, and sodium lauryl sulfate (SLS) at 0.5%, 0.5% and 0.1% (w/w), respectively, was used as a stabilizer solution [218]. When compared to raw artemether, the water solubility of artemether nanocrystal was improved sixfold and thus increased its bioavailability. This was attributed to the increased surface area of the nanocrystal [218]. The physical stability of the nanocrystal over 90 days was shown to be exceptional at really low temperatures (2°–8 °C), at room temperature (25 °C), and in acidic conditions (pH 2). Artemether nanocrystals had the greatest reduced parasitaemia, 89%, compared to the unprocessed and marketed tablets which reduced parasitaemia by 25% and 45%, respectively [218].

The same study was conducted for lumefantrine with the aim of formulating smart nanocrystals of lumefantrine, using a stabilizer solution containing 0.5% w/w of PVK-K30 and 0.5% w/w SLS. Lumefantrine nanosuspension was prepared by wet milling using Dena® DM-100. The lumefantrine nanocrystals were found to be stable under the same conditions as artemether nanocrystals. Plasmodial parasitaemia was also greatly decreased by 82% by the nanocrystal, which was 2.3-fold and 4.3- fold greater than the market formulation and unprocessed lumefantrine, respectively. Both the in vivo and in vitro studies showed that the antimalarial potential of the nanocrystal was significantly enhanced compared to its counterparts. Shah et al. then concluded that the enhanced efficacy was due to the increased surface area and the rapid dissolution [219].

Li et al. used zeolitic imidazolate framework-8 (ZIF-8) to load dihydroartemisinin. ZIF-8 had good biocompatibility, a high drug encapsulation efficacy, and a controllable drug release. This nanoparticle showed no toxicity, and the magnetic resonance imaging contrast effect was enhanced [220].

In another study, Shah et al. formulated lyophilized nanosuspensions of lumefantrine to address the drug’s aqueous solubility and oral bioavailability. For the preparation of the nanosuspension, a complex containing lumefantrine, soya lecithin, and PVP-K30 was generated in the ratio of 1: 4: 1. This complex is soluble in water (0.921 mg/mL) and converts the crystalline drug to amorphous form. Lumefantrine nanosuspensions were prepared using anti-solvent precipitation and ultra-sonication techniques using chloroform as the organic solvent, which was selected due to the high solubility of drug (106 mg/mL). The lyophilized lumefantrine was physically stable for 3 months and no significant change was observed at 2–8 °C and around 25 °C. However, when the sample was stored at ~ 40 °C, a slight increase in particle size was observed. This was attributed to the elevated kinetic energy imparted by temperature leading to the collision and enhanced likelihood of aggregation. In comparison to pure lumefantrine and the dry syrup marketed formulation of lumefantrine, lumefantrine nanosuspension exhibited a higher drug release rate in vitro. This was attributed to its low solubility in water and hydrophobic properties. Within the first 15 min, the cumulative lumefantrine released from the nanosuspension was 90%, compared to 10% for free lumefantrine and > 60% for the marketed formulation. This may be because the lumefantrine in nanosuspension has smaller particle sizes, which increases its surface area. Additionally, the co-grinding of the stabilizer with the lumefantrine may increase the surface wetting of the nanosuspension, which increases surface wetting and consequently, increase dissolution. Particles in the nanosuspension have more chances of interaction with chloroform due to small particle size and may lead to solubility. The nanosuspension formulation demonstrated a ~ eightfold increase in drug release compared to the pure lumefantrine. This showed that nanosuspensions can be used to successfully increase the dissolution rate of the drug. Furthermore, lumefantrine-nanosuspension was active against *P. falciparum* at exceptionally low concentrations in vitro. The formed nanosuspension has increased solubility, therefore, it was concluded that the nanosuspension can be easily administered by the pediatric and geriatric population, as well as anybody who has difficulty swallowing oral solid dosage forms such as tablets [221].

### Stimuli-responsive drug delivery

Stimuli-responsive systems address the difficulty of successfully releasing the drug in a controlled fashion to the target cell while preserving the drug in the extracellular environment [222]. These recognize certain microenvironmental stimuli and respond by emulating the reaction of living organisms. This approach requires the use of biocompatible materials that undergo specific protonation, hydrolytic cleavage, molecular assembly, and conformational changes in response to the stimuli. Endogenous and exogenous stimuli evoke drug release by the carrier. Exogenous stimuli can be introduced at the desired spatial location and function as extracellular stimuli for cells. These are external factors such as light, magnetic, and electric field, ultrasound, and temperature, which is usually 40–42 °C for diseased tissue [222–227]. Changes in redox, pH, enzyme concentration, and gradient are all endogenous stimuli that have been associated with disease development in the body. These regulate drug distribution and release from nanocarriers [227, 228].

#### Redox-responsive drug delivery systems

Redox-responsive delivery systems are frequently utilized to decrease the toxicity and adverse effects of the drug, while enhancing the drug’s concentration and efficacy, and rapid drug delivery at the targeted cell [229, 230].

In a study by Long et al., a redox-sensitive polymeric micelle system was formed to enhance artesunate activity [225]. The micelles were formed by the self-assembly of K5 capsular polysaccharide-SS-D-α-tocopheryl succinate (K5-SSTOS, KSST) copolymer. In vitro studies showed that the micelle reduced the side effects of artesunate, as the blank micelle was almost non-toxic, and also kept cell viability > 90% even after being incubated for over 24 h. The low hemolysis ratio and low cytotoxicity demonstrated that the micelle can be used as a nanocarrier for enhancing artesunate activity and that this enhanced activity was achieved by responding to redox-sensitive drug release in the target site [225].

The reduction-sensitive amphiphilic poly (2-methyl-2-oxazoline) PMOXA-graft(SS)-PCL copolymer was synthesized, and its application as a smart nanocarrier for Nile Red was assessed [231]. This forms a polymer brush that is dense, highly hydrated, and colloidally stabilizes the encapsulated particle and protects it from non-specific protein adsorption and biological response. Nile Red is a hydrophobic fluorophore dye molecule used as a drug model for poorly water-soluble drugs [232, [233]. Nile Red was encapsulated in the core of the reduction-sensitive amphiphilic copolymer, and after 2 h of incubation of the encapsulated dye with a mixture of malaria parasite-infected erythrocytes and erythrocytes, an intracellular parasite staining was observed. This demonstrated that the drug model was passively delivered to the infected erythrocytes using the copolymer. When compared to erythrocytes, the dye was identified in the infected erythrocyte more frequently. It was concluded that the use of targeting ligands such as antibodies will further increase the targeting of infected erythrocytes. The rapid delivery of the model drug to the target site without the use of any targeting ligands showed that PMOXA-coated nanoparticles can potentially be used for passive targeted antimalarial delivery to plasmodium infected erythrocytes [231].

#### pH-responsive drug delivery systems

pH-responsive materials react to changes in the pH of their environment. When in contact, the presence of specific functional groups in the chain and the pH of the medium causes the polymer to either inflate or collapse. These materials are either acidic or alkaline [190, 234].

Dendrimer polymers are pH-stimulated and are used in drug delivery systems because they have a large surface area, good solubility, can enhance drugs solubility and bioavailability when formulated and are non-immunogenic and non-toxic. The large surface area of the dendrimers prevents them from being filtered out by the kidney, resulting in increased retention in the body. Additionally, their excellent structural uniformity and a high degree of branching and functionality render them simple to manipulate for drug delivery systems [190, 235].

Dendrimers have scaffolding characteristics and operate as nanoscale containers. These feature structural parts that allow them to function as nanoscale containers, such as a focal core with either a single atom or a group of atoms with identical chemical functions. Due to the unique nanoenvironment, this encapsulates chemicals with unrivalled characteristics. Second, building blocks with several interior layers composed of repeating units that provide a flexible area within the dendritic building blocks that contain tiny guest molecules. Finally, there is the multivalent surface, which interacts with the external environment to contain a wide range of functions [236, 237]. They bind to the drug by either covalent bonds, which can be cleavable, or non-covalent bonds. Targeted drug delivery cannot be done if the interaction occurs via cleavable bonds, but this stimulates the excretion of the dendrimer [235, 238].

Bhandra and Jain explored the use of uncoated and chondroitin sulfate A (CSA)-coated PEGylated poly-L-lysine-based dendrimers for controlled sustained delivery of intravenously administered chloroquine phosphate. CSA-coated dendrimers were found to have increased size and drug loading capacity, as well as decreased drug release. The CSA coating of the dendrimer masked and reduced the hemolytic toxicity and cytotoxicity of chloroquine phosphate [239]. Similarly, the release of artemether was prolonged by solubilizing it in PEG-lysine-type dendritic peptide-based nanoparticulate carriers for up to 1–2 days. When conjugated with CSA, the drug loading capacity and duration of release were increased by two–threefolds. Conjugation, in vitro, helped clear the circulating ring and trophozoite stages of *P. falciparum.* Both uncoated and coated systems had prolonged artemether release in vivo. From both studies, it can be deduced that this system can act as sustained release circulating nanocarriers for controlled delivery of antimalarials [239, 240].

### Carbon-based drug delivery systems

Nanotechnology makes use of carbon nanomaterials because of their distinctive characteristics and flexible structure. The size of carbon nanomaterials ranges from 1 to 100 nm [241]. Due to their nano-size, the properties of carbon nanomaterials are dependent on their atomic structures and interaction with other molecules [242]. Examples of carbon nanomaterials are fullerenes, carbon dots, graphene, and carbon nanotubes.

#### Fullerenes

Fullerenes are molecules composed of carbon atoms only and are one of carbon’s allotropes. These are nano-sized molecules (1 nm), and structurally, they can be spherical, tubular, or ellipsoidal [241, 243]. Due to their size, fullerene derivatives such as C_60_, C_70_, and C_20_ can penetrate the plasma membrane [244]. C_60_ is the common fullerene derivative and contains fused rings of 20 hexagons and 12 pentagons [243, 245].

Due to the solubility of fullerenes in organic solvents, they can readily accept electrons. These are insoluble in aqueous media and aggregate readily. However, the alteration of fullerenes by the addition of hydroxyl groups (fullerenols) improves their aqueous solubility. These do not act as electron-rich aromatic systems but as electron-deficient alkenes and they can conduct electricity [240, 246, 247]. The properties of fullerene derivatives, such as their spherical shape, antioxidant activity, loading efficiency, low side effects, and capacity to sustain drug release make them ideal for drug delivery [248].

Novir and Aram, investigated the adsorption of chloroquine by pristine C_60_ fullerene and found that upon this non-covalent interaction, both C_60_ and chloroquine were unchanged. The pristine C_60_-chloroquine complex, an electrically harmless interaction, was more stable and presented a lower solvation energy. Solvation energy is indirectly proportional to solubility. Thus, the solubility of chloroquine was enhanced in the complex. In the same study, aluminum-doped C60 formed a complex with chloroquine. The doped displayed high binding energy which resulted in a robust interaction with chloroquine. In contrast to the pristine complex, the doped C_60_ showed substantially lower solvation energy, indicating that the drug’s solubility was enhanced even more. This indicated that the C_60_ fullerenes can be used as nanocarriers [248].

#### Carbon nanotubes

Carbon nanotubes (CNTs) are large cylindrical molecules consisting of Sp^2^ hybridized carbon-atom hexagons with a hydrophobic surface. These carbon allotropes are arranged in a helical fashion about the needle axis [249–251]. They are made up of rolled-up graphene sheets. CNTs are classified based on the number of layers, as either single-walled CNTs (SWCNTs) or multiwalled CNTs (MWCNTs) which have 2 or more layers 0.34 nm apart [252–255]. CNTs are usually synthesized using low-temperature chemical vapor deposition methods because the nanotube length, diameter, density, purity, and orientation can be effectively controlled at low temperatures [252].

CNTs are non-biodegradable and toxic, thus, they can be functionalized with basic macromolecules or organic molecules to make them less toxic and more biocompatible. These are functionalized either covalently or non-covalently. Non-covalent functionalization occurs when CNTs are coated with amphiphilic surfactant molecules, whereby the hydrophobic part of the adsorbed molecules interacts with the walls of the nanotube either within or outside the CNT, via van der Waals, hydrophobic and π-π interactions, [256–258]. Covalent functionalization occurs when chemical molecules covalently bond to the surface of CNTs and share electron pairs. Typically, strong acids oxidize CNTs to form carboxyl groups at the tips of CNTs [257, 259].

Their properties include high drug loading capacity, the ability to release drugs at the target site, high surface area, high chemical, and thermal stability, enhanced conductivity, the ability to be easily functionalized, their needle-like structure, and their flexible interaction with their cargo. These properties, in addition to the antioxidant activities, enable CNTs to penetrate cell membranes, carry multiple drugs at high density, and reduce the adverse effects of drugs while effectively delivering them to cells [254, 256, 257, 260].

A curcumin-loaded SWCNT was used to circumvent the poor solubility of curcumin and evaluate its stability using ultrasonication. Factors such as the large surface area of the SWCNT and the molecular structure of curcumin, the use of the ultrasonicator, and van der Waals forces between the SWCNT and curcumin enhanced the loading efficiency of curcumin to 94%. The formulated SWCNT increased the solubility of curcumin to 1.88 mg/mL from ~ 0.007 mg/mL in aqueous media and also protected it from degradation. In vitro*,* 5% of the curcumin was hydrolyzed and biotransformed. Indicating that the formulated SWCNT can increase the stability of curcumin by reducing its biodegradation. In addition, 95% of curcumin from the formulated SWCNT was released into the medium after 1 h and the cumulative release rate slightly decreased after 12 h [260].

In another study, SWCNTs were used as nanocarriers to investigate the delivery of artemisinin and its derivatives against the translationally controlled tumor protein of *P. falciparum* utilizing a molecular docking simulation. The docking simulation showed that artemisinin and its derivatives, artemether, and artesunate, had molecular docking scores of -32.66, -26.99, and -14.29, respectively. The docking score predicts the binding affinity of the docked target and ligand. These scores indicate that there’s a strong binding between the drug and the tumor protein, and thus favorable molecular interactions at the active site of the enzyme. It was deduced that artemisinin and its 2 derivatives serve as good inhibitors using SWCNTs as carriers [261].

### Target-specific nanomaterials

When enhancing permeability and retention is not appropriate or is ineffective, a nanoparticle delivery mechanism must be targeted for selective distribution to pathogenic sites. Targeted drug delivery is defined as a method of administering the drug moiety precisely to the organ, cellular, and subcellular level of the targeted body location to avoid unintended harmful effects of traditional drug administration and lower the dosage needed for therapeutic efficacy [146, 262, 263]. Therapy of diseases such as cancer and malaria necessitate active cell targeting. Exogenous stimuli can be used to stimulate drug release by nanoparticles.

Passive targeting delivery targets systemic circulation, which occurs as a result of the body’s natural response to the physicochemical characteristics of the drug, or the carrier system and it is based on the enhanced permeability and retention effect. Active targeting, on the other hand, is based on the direct delivery of the drug to the specific diseased area in the body and it mostly depends on the biological contact between the ligands associated with nanoparticles and the target cell [262, 264]. In contrast to passive targeting, which uses more common nanocarriers like liposomes, active targeting makes use of nanocarrier surfaces modified with specific ligands such as proteins, peptides, carbohydrates, or antibodies. The nanocarriers used in targeted delivery with a ligand must have functional groups that can be conjugated to a targeting desired ligand, as this allows for selective delivery of the drug. Site-specific ligands are commonly coupled with liposomes to achieve active targeting [11, 262, 263, 265].

#### Antibody-specific targeted nanoparticles

Since antibodies are highly specific for their target antigens, they are commonly used as target molecules for liposomes. Immunoliposomes are formed by coupling antibodies to the liposomal surface, they enable active tissue targeting through binding to tumor cell-specific receptors [266, 267]. Targeted liposomes were used for the delivery of antimalarial lipids. When integrated into a liposome formulation, the lipid maleimidophenyl butyramide phosphoethanolamine (MPB-PE), which is used to covalently cross-link antibodies to liposomes through thioether linkages, significantly exhibited antiparasitic effect by inhibiting the growth of *P. falciparum *in vitro. This suggested that lipids entered the cell and reached the pathogen when the liposome randomly interacted with plasmodium-infected erythrocytes [10]. In addition, heparin was used as an antimalarial drug and a targeting element for primaquine-loaded liposomes. In *P. falciparum* cultures, heparin electrostatically adsorbed onto passively charged primaquine loaded liposome, increased the activity of encapsulated drug by threefold without penetrating the healthy erythrocytes. The parasiticidal activity was a synergistic activity of free heparin as an antimalarial drug and of liposome-bound heparin as the targeting element for encapsulated primaquine [7, 10].

Chloroquine was encapsulated in agmatine-containing poly(amidoamine) polymer (AGMA1) to selectively deliver it to the plasmodium-infected erythrocytes to limit parasite resistance. Since AGMA1 is capable of entering plasmodium-infected erythrocytes during the trophozoite stage of the malaria life cycle, it is thought to have an antimalarial effect. In vitro*,* the growth of *P. falciparum* was inhibited by AGMA1, which exhibited an IC_50_ of 13.7 µM. With a radius of ~ 7 nm, AGMA has a significant chloroquine loading capacity. In vivo toxicity assay showed that AGMA1, whether loaded or free, did not have any observable adverse effects. 0.8 mg/kg of free chloroquine and chloroquine-loaded AGMA1 was intraperitoneally administered over 4 days. The chloroquine-loaded AGMA1 showed a high concentration peak 1.5 h after injection and was completely dissolved within 3 h. After 4 days, a suppression test revealed that ~ 100% of the parasite was removed by the chloroquine-loaded AGMA1, compared to 50% parasitaemia eliminated by the free chloroquine. This was attributed to the polymers specifically targeting the infected erythrocytes without internalizing in healthy erythrocytes [268].

In another study, quantum dot-loaded liposomes were functionalized with specific half antibodies against *P. falciparum* infected erythrocytes. The targeted immunoliposomes recognized and penetrated the infected erythrocytes’ membrane, and there released the ‘cargo’ [10].

*Plasmodium* parasites use erythrocyte receptor entry points to initiate the blood stage of malaria [269]. To circumvent this, Urban et al. used BM1234-mediated targeted delivery of immunoliposome-encapsulated drug to target the *plasmodium* antigens. It was found that the antibody BM1234 was only specific for plasmodium-infected erythrocytes infected by the trophozoite and schizont, and not the ring-stage plasmodium-infected erythrocytes. Owing to this, it was speculated that trophozoites would be a better target for BM1234-mediated targeted delivery of immunoliposome-encapsulated drugs than rings. Although the encapsulating BM1234 immunoliposomes enhance drug efficacy by 10-folds and deliver their contents to all plasmodium-infected erythrocytes in the sample, these cannot eliminate all *Plasmodium* parasites [267].

A summary of the nanovehicles developed for the prevention and treatment of malaria is provided in Table 3.

**Table 3:** Nanoparticles and their effect on antimalarial drugs

Nanoparticle	In vivo/In vitro	Loaded drug	Notable outcomes	References
Liposome	In vivo*. *In vitro	Artemether-Lumefantrine	Prolonged drug retention and availability No renal and hepatic toxicity	[18]
	In vivo	Artesunate	Sustained release at the target site	[147]
	In vivo	Chloroquine	Sustained release at the target site No toxic effects	[148]
Niosome	In vivo	Primaquine-curcumin	100% enhanced antimalarial activity Reduced rate of recrudescence Enhanced therapeutic efficacy No renal and hepatic toxic effect	[19]
	In vitro	Artemether	Good entrapment efficacy Higher antimalarial activity	[155]
Ethosome	In vitro	Artesunate Febrifugine	Enhanced release rate Enhanced antimalarial efficiency Enhanced accumulative skin permeation	[156]
SLN	In vitro	Artemether	enhanced stability and intestinal permeability	[167]
	In vitro	Chloroquine	Enhanced efficacy	[168]
	In vivo	Dihydroartemisinin	Enhanced drug efficacy Enhanced encapsulation efficacy	[169]
	In vitro	Primaquine	Higher chemosuppression, increased antimalarial activity	[170]
	In vitro	Artesunate	enhanced the aqueous solubility, bioavailability, and intestinal permeability	[171]
Nanosponge	In vitro	Chloroquine	Improved drug activity and uptake	[186]
Hydrogel	In vitro	Chloroquine phosphate-curcumin	Drugs are released at different times to overcome drug resistance	[13]
Cyclodextrin	In vitro	Artemether	Enhanced water solubility and bioavailability	[209]
		Primaquine	Reduced drug release and increased drug activity	[211, 212]
Nanosuspension	In vitro	Lumefantrine	Higher drug release rate Increased dissolution	[218]
Micelle	In vitro	Artesunate	Increased activity of drug Increased release at the target site	[ 225]
Dendrimer	In vivo	Chloroquine phosphate	Reduced hemolytic toxicity and cytotoxicity of drug. Decreased drug release	[239]
	In vitro	Artemether	Prolonged release of the drug Controlled drug delivery	[239, 240]
Fullerenes	In vitro	Chloroquine	Increased drug solubility	[248]
SWCNT	In vitro	Curcumin	Enhanced drug solubility in aqueous media Reduced biodegradation of drug	[260]
	In vitro	Artemisinin, artemether, artesunate	Increased activity of the drug	[261]
AGMA1	In vitro	Chloroquine	Removed 100% of the parasite	[267]

## Conclusion

Malaria is a very deadly parasitic disease, mainly afflicting pregnant women and children under the age of 5. Notwithstanding that there are relatively vast options for treating malaria, there are reports from some areas reporting that the *Plasmodium* has developed resistance against therapeutics such as chloroquine due to overuse of antimalarial drugs as prophylaxis and poor patient adherence among others [2, 6]. Although, ACT drugs have shown good activity, factors such as their unpalatability, poor aqueous solubility and adverse effects have led to poor patient adherence and eventually, possible parasite resistance. Moreover, these drugs undergo extensive first-pass metabolism, demonstrate no-specificity and are absorbed by various cells in the body; thus, their antimalarial activity is lower than what it could potentially be.

Nanomaterials are capable of circumventing aforementioned drawbacks. The nanoscale dimensions of the particles increase their surface area-to-volume ratio. Thus, nanoparticles can increase the solubility and hence the bioavailability and efficacy of drugs. Nanomaterials also have the ability to release the drug at the target site. A controlled targeted release of drugs aid in eliminating the cytotoxicity of drugs, while simultaneously increasing their antimalarial activity and parasitaemia reduction since it bypasses the metabolism.

Nanomaterials do also have the advantages in further development of vaccines treatment modalities but use of additive techniques. For instance, the development of stimuli-responsive delivery systems has the potential to provide the possibility of spatiotemporal drug delivery facilitating the potential for direct to parasite and on-demand therapeutic options. The development of nanovehicle-in-microneedles as well as nanovehicle-in-nanofiber have the potential to unlock more possibilities in transdermal treatment of malaria. These two technologies could revolutionize the way malaria is treated and prevented.

Despite these exciting potential applications, nanomaterial use for malaria treatment and prevention is still in its early stages. This is primarily due to a lack of thorough investigations, as the majority of studies concentrate on testing nanovehicles against a single Plasmodium species from a single source (typically laboratory strains), rather than taking into account a comprehensive assessment using different species from multiple origins (e.g., clinical isolates). To fully realize the potential of nanotechnology to prevent and treat important prerequisites, viz. production cost, social impact as well as complementary studies including physicochemical stability, scaling up, sterilization process, toxicology, oral and transdermal delivery are required. This will further boost the translational development of anti-malarial medications toward clinical use.

## Data Availability

All data generated or analyzed during this study are included in this published article.

## Abbreviations

ACT: Artemisinin-based combinational therapy
CNT: Carbon nanotubes
CSA: Chondroitin sulfate A
CSP: Circumsporozoite protein
DEET: Diethyltoluamide
HPβCD: 2-Hydroxypropyl-β-cyclodextrin
LBDDS: Lipid-based drug delivery systems
MWCNT: Multiwalled CNTs
NLC: Nanostructure lipid carrier
PLA: Poly(lactic acid)
PLGA: Poly(lactic-coglycolic acid)
PMOXA: Poly (2-methyl-2-oxazoline)
SEDDS: Self-emulsifying drug delivery system
SMEDDS: Self-micro-emulsifying drug delivery system
SLN: Solid lipid nanoparticles
SWCNT: Single-walled CNTs
